# Downregulated *MTAP* expression in myxofibrosarcoma: A characterization of inactivating mechanisms, tumor suppressive function, and therapeutic relevance

**DOI:** 10.18632/oncotarget.2552

**Published:** 2014-10-24

**Authors:** Chien-Feng Li, Fu-Min Fang, Hsing-Jien Kung, Li-Tzong Chen, Jun-Wen Wang, Jen-Wei Tsai, Shih Chen Yu, Yu-Hui Wang, Shau-Hsuan Li, Hsuan-Ying Huang

**Affiliations:** ^1^ Department of Pathology, Chi-Mei Medical Center, Tainan, Taiwan; ^2^ Department of Biotechnology, Southern Taiwan University of Science and Technology, Tainan, Taiwan; ^3^ National Institute of Cancer Research National Health Research Institutes, Tainan, Taiwan; ^4^ Institute of Clinical Medicine, Kaohsiung Medical University, Kaohsiung, Taiwan; ^5^ Departments of Radiation Oncology, Kaohsiung Chang Gung Memorial Hospital and Chang Gung University College of Medicine, Kaohsiung, Taiwan; ^6^ Institute of Molecular and Genomic Medicine, National Health Research Institutes, Tainan, Taiwan; ^7^ Department of Internal Medicine and Cancer Center, Kaohsiung Medical University Hospital, Kaohsiung Medical University, Kaohsiung, Taiwan; ^8^ Orthopedic Surgery, Kaohsiung Chang Gung Memorial Hospital and Chang Gung University College of Medicine, Kaohsiung, Taiwan; ^9^ Department of Anatomic Pathology, E-Da Hospital, Kaohsiung, Tawian; ^10^ Department of Pathology, Kaohsiung Chang Gung Memorial Hospital and Chang Gung University College of Medicine, Kaohsiung, Taiwan; ^11^ Institute of Biosignal Transduction, National Cheng Kung University, Tainan, Taiwan; ^12^ Department of Internal Medicine, Division of Oncology, Kaohsiung Chang Gung Memorial Hospital and Chang Gung University College of Medicine, Kaohsiung, Taiwan

**Keywords:** myxofibrosarcoma, MTAP, homozygous deletion, methylation

## Abstract

Myxofibrosarcomas are genetically complex and involve recurrently deleted chromosome 9p, for which we characterized the pathogenically relevant target(s) using genomic profiling. In 12 of the 15 samples, we detected complete or partial losses of 9p. The only aggressiveness-associated, differentially lost region was 9p21.3, spanning the potential inactivated methylthioadenosine phosphorylase (*MTAP*) that exhibited homozygous (4/15) or hemizygous (3/15) deletions. In independent samples, *MTAP* gene status was assessed using quantitative- and methylation-specific PCR assays, and immunoexpression was evaluated. We applied MTAP reexpression or knockdown to elucidate the functional roles of MTAP and the therapeutic potential of L-alanosine in MTAP-preserved and MTAP-deficient myxofibrosarcoma cell lines and xenografts. MTAP protein deficiency (37%) was associated with *MTAP* gene inactivation (*P* < 0.001) by homozygous deletion or promoter methylation, and independently portended unfavorable metastasis-free survival (*P* = 0.0318) and disease-specific survival (*P* = 0.014). Among the MTAP-deficient cases, the homozygous deletion of *MTAP* predicted adverse outcome. In MTAP-deficient cells, MTAP reexpression inhibited cell migration and invasion, proliferation, and anchorage-independent colony formation and downregulated cyclin D1. This approach also attenuated the tube-forming abilities of human umbilical venous endothelial cells, attributable to the transcriptional repression of MMP-9, and abrogated the susceptibility to L-alanosine. The inhibiting effects of MTAP expression on tumor growth, angiogenesis, and the induction of apoptosis by L-alanosine were validated using MTAP-reexpressing xenografts and reverted using RNA interference in MTAP-preserved cells. In conclusion, homozygous deletion primarily accounts for the adverse prognostic impact of MTAP deficiency and confers the biological aggressiveness and susceptibility to L-alanosine in myxofibrosarcomas.

## INTRODUCTION

Myxofibrosarcoma is characterized by the multinodular growth of spindle to polygonal sarcoma cells within variably myxoid stroma containing long curvilinear vessels [1, 2]. Clinically, increased tumor grades and stages are frequently observed in myxofibrosarcomas after local recurrence, and may cause metastatic diseases [1–4]. However, histological evaluation is insufficiently satisfactory to predict aggressiveness of individual cases, indicating the need to elucidate the pathogenesis of myxofibrosarcoma [2, 3].

Genetically, complex karyotypic changes are characteristic of myxofibrosarcomas [4, 5] and a recent large-scale genomic study indicated that a nonrandom loss of chromosome 9, and particularly 9p, occurs in myxofibrosarcomas [5]. By using ultrahigh-resolution array comparative genomic hybridization (aCGH), we previously profiled the global copy-number alterations (CNAs) in myxofibrosarcoma specimens and cell lines and characterized *SKP2* on 5p and *CDK6* and *MET* on 7q as amplified oncogenes of pathogenic relevance [6–8]. Regarding DNA losses, chromosome 9p was the most frequently lost chromosomal arm in myxofibrosarcomas [5], prompting the search for potential tumor suppressor gene(s) underlying this selection pressure for the loss of 9p. We characterized methylthioadenosine phosphorylase (*MTAP*) on 9p21.3 because whether the tumor-suppressive role of this polyamine metabolism-regulating enzyme is independent from the frequently co-deleted *CDKN2A* and *CDKN2B* genes still remains debated [9–12].

In this study, MTAP protein deficiency in myxofibrosarcomas was associated with a poor prognosis and inactivated *MTAP* gene, caused by either homozygous deletion or promoter methylation. Functionally, MTAP deficiency yielded increased aggression in myxofibrosarcoma cells. By restricting the adenosine monophosphate (AMP) supply [13, 14], L-alanosine induced prominent apoptosis in the MTAP-deficient myxofibrosarcoma cells and derived xenografts. Collectively, the mechanistic and clinical evidence reinforces *MTAP* as a functional tumor suppressor gene exhibiting prognostic and therapeutic relevance in myxofibrosarcomas.

## RESULTS

### Genomic profiling revealed recurrent 9p loss

**Figure 1:**
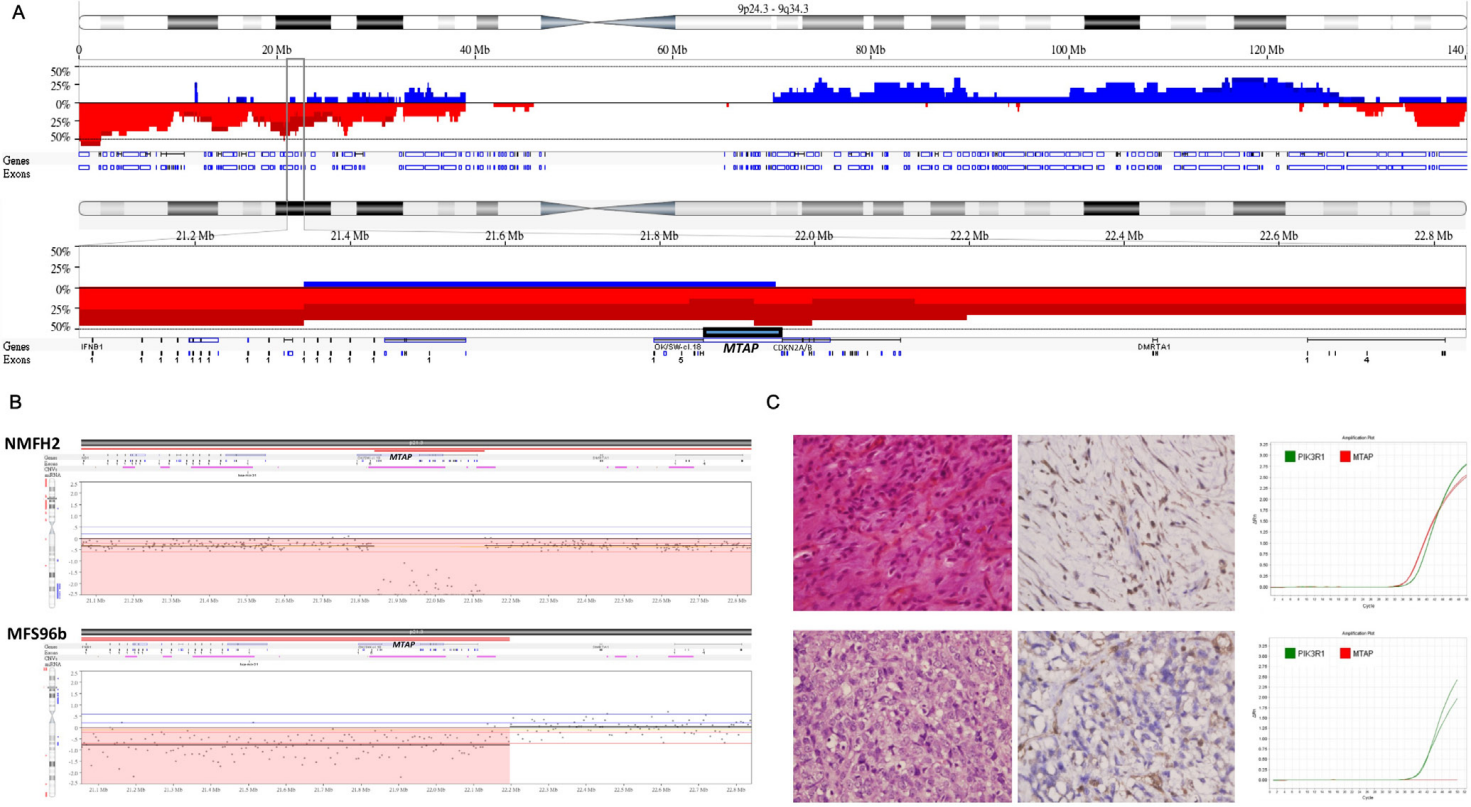
*MTAP* homozygous deletion in myxofibrosarcoma **(A)** Referring to chromosome 9 cytoband, genomic profiling exhibits near arm-level losses of 9p clustered in five major deletion cores *(top)*. Within the middle deletion core (9p22.2-p21.1), homozygous deletions frequently involves the aggressiveness-associated 9p21.3 region as delineated by the rectangle *(middle)*. At higher magnification, the genomic locus of *MTAP* gene in the 9p21.3 region is illustrated *(bottom)*. Frequency plots of the CNAs are expressed in blue (gain) or red (loss). **(B)** The close-up view of representative myxofibrosarcoma cell line (NMFH-2) and tissue (MFS96b) samples shows the homozygous deletion of the *MTAP* gene. Intratumoral nonneoplastic cells in MFS96b account for the less prominent loss of *MTAP* copies. **(C)** Upper: A Grade 1 myxofibrosarcoma stained with hematoxylin-eosin *(left)* shows diffused MTAP expression *(middle)* and a preserved *MTAP* gene, shown as the red curve in a quantitative DNA-PCR *(right)*. Lower: A Grade 3 myxofibrosarcoma stained with hematoxylin-eosin *(left)* shows MTAP-deficient sarcoma cells *(middle)* and the nondetectable *MTAP* gene *(right)*.

Chromosomal imbalances of varying degrees were detected in all samples subjected to aCGH profiling, indicating more recurrent deletions than gains, and exhibiting characteristically high genomic complexity. According to filter criteria, Nexus software revealed recurrent DNA gains in 211 chromosomal regions spanning 4577 genes in all of the genomes. However, 235 chromosomal regions were nonrandomly lost, involving 7871 named genes. In the long arm of chromosome 9, the copy number alterations were predominantly of DNA gains, except for the 9q34 region that exhibited DNA losses. In contrast, the complete or partial losses of 9p were detected in 12 of the 15 samples, and five major deletion cores on 9p, recurrent in ≥ 20% of samples tested, were interspersed with short stretches of DNA gains (Figures 1A, B, Supplementary Table S1). Within the 9p22.2-p21.1 deletion core, the sole differentially deleted, aggressiveness-associated region on 9p was narrowed down to 9p21.3 (*P* = 0.0454). This result indicated the implication of 9p21.3 in the myxofibrosarcoma progression, in which *MTAP* and *CDKN2A*/*CDKN2B* were homozygously deleted in 4 and 4 samples and hemizygously deleted in 3 and 3 samples, respectively (Table S2).

### Associations of MTAP immunoexpression with clinicopathological and gene statuses in primary myxofibrosarcomas

The MTAP immunostain of 87 independent primary myxofibrosarcomas (Figure 1C) demonstrated an aberrant MTAP deficiency in 32 cases (37%). *MTAP* gene dosage was successfully determined in 79 cases, 20 of which (25.3%) exhibited homozygous deletion at an *MTAP/PFKL* ratio of < 0.2 (Table 1, Figure 1C). Because 13 of the 29 MTAP protein-deficient tumors were not homozygously deleted at the *MTAP* gene (Table 1), methylation-specific PCR was adopted to examine whether promoter hypermethylation alternatively caused protein loss, and 10 of these 13 cases were hypermethylated at the *MTAP* promoter (Table 1, Figure S1A). MTAP protein deficiency was strongly related to inactivated *MTAP* genes (*P* < 0.001, Table 1), either by homozygous deletion or promoter methylation. Regarding the status of MTAP protein expression and promoter methylation, no significant difference was detected in the clincopathological features, including the tumor grading and staging. By contrast, *MTAP* homozygous deletion was significantly correlated with high histological grades (*P* = 0.006, Figure 1C, Table 1) and a high mitotic rate (*P* = 0.011, Figure S1B), and marginally correlated with advanced clinical stages (*P* = 0.097).

**Table 1:** Associations of clinicopathological features with MTAP immunoexpression and gene status in primary myxofibrosarcomas

MTAP Expression (n=87)			p-value	MTAP Gene (n=79)		p-value
	Deficient	Positive		HD	Non-HD	
**Sex**			1.000			0.936
Male	18	31		8	23	
Female	14	24		12	36	
**Age**			1.000			0.854
<60 years	12	21		23	7	
≥60 years	20	30		37	13	
**Location**			0.799			0.908
Extremity	23	42		15	45	
Axial	9	13		5	14	
**Tumor necrosis**			0.483			0.115
<10%	19	38		41	10	
>=10%	13	17		18	10	
**FNCLCC grade**			0.534			0.006*
grade 1	13	25		30	4	
grade 2	12	23		24	9	
grade 3	7	7		5	7	
**AJCC stage**			0.763			0.097
Stage 1	6	14		16	1	
Stage 2	12	19		21	9	
Stage 3	13	20		20	10	
**Tumor size^&^**	7.314±6.528	6.347±3.701	0.633	6.600 4.808	7.311 5.551	0.612
**Mitotic rate^&^**	12.81±13.143	10.33±1.695	0.585	8.780±9.180	19.050±15.408	0.011*
***MTAP*** gene status			<0.001			
Aberration (*HD, PM*)	26 (*16,10*)	4 (*4,0*)				
No aberration (*no HD or PM detected*)	3	46				

HD: homozygous deletion, PM: promoter methylation,

* :Statistically significant,

& :t-test,

### Survival analyses

**Figure 2:**
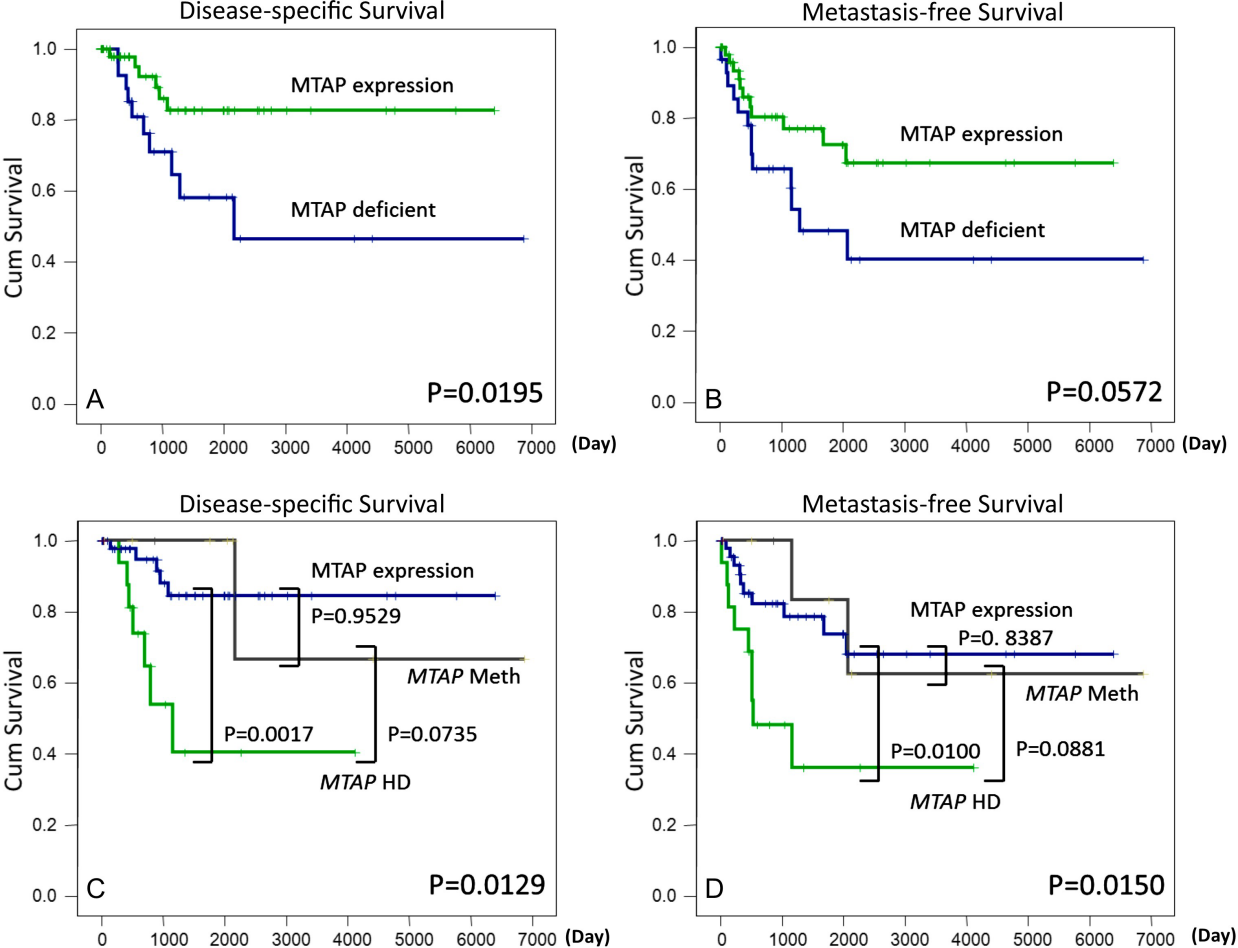
MTAP protein deficiency predicts adverse disease-specific **(A)** and metastasis-free survival **(B)** rates. Myxofibrosarcomas involving homozygously deleted *MTAP* genes yield significantly poor outcomes for both survival endpoints **(C, D)** and MTAP-expressing and *MTAP*-methylated cases did not differ prognostically.

Univariate correlations of the clinical outcome with various clinicopathological, immunohistochemical, and molecular parameters are shown in Table S3 and Figure 2. MTAP protein deficiency was a significant adverse prognosticator of an adverse DSS (*P* = 0.0195, Figure 2A) and was marginally predictive of a short MFS (*P* = 0.0572, Figure 2B). Regarding various mechanisms regulating MTAP expression, *MTAP*-homozygously deleted myxofibrosarcomas behaved aggressively and exhibited significantly shorter DSS (*P* = 0.0129, Figure 2C) and MFS (*P* = 0.0150, Figure 2D) values than did the cases lacking homozygous deletion. Among the nonhomozygously deleted cases, no difference in prognosis was observed between the MTAP-expressing and MTAP-hypermethylated cases. Compared with the homozygously deleted cases, the MTAP-deficient tumors caused by methylation exhibited a trend toward more favorable outcomes (*P* = 0.0735, Figure 2C; *P* = 0.0881, Figure 2D). These results suggested variable implications of distinct inactivating mechanisms in MTAP-deficient tumors. In the multivariate analysis (Table S4), MTAP protein deficiency was the single independent prognosticator of an inferior DSS (*P* = 0.0140, hazard ratio = 4.020) and, along with high histological grades, could predict inferior MFS (*P* = 0.0318, hazard ratio = 2.527).

### Tumor suppressive functions of MTAP in myxofibrosarcoma

**Figure 3:**
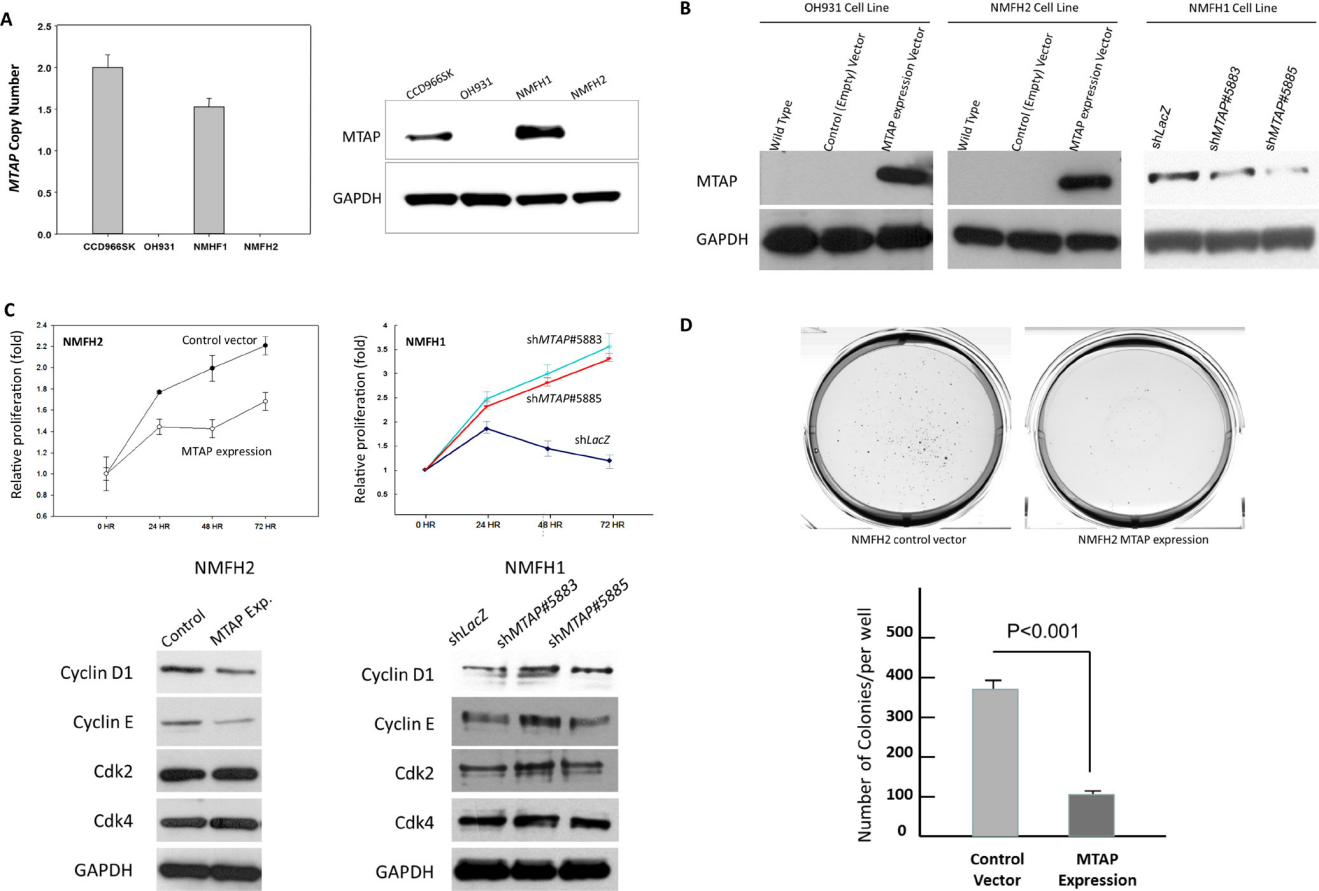
*In vitro* growth-inhibiting function of MTAP linked to downregulated cyclin D1 **(A)** Compared with reference CCD966SK fibroblasts, the OH931 and NMFH-2 cell lines were homozygously deleted at the *MTAP* gene; no DNA copy was detected using real-time PCR *(left)* and the western blots exhibited no MTAP expression *(right)*. Although the *MTAP* gene dosage was slightly decreased, a western blotting assay revealed MTAP-expressing NMFH-1 cells. **(B)** Stable exogenous MTAP reexpression was achieved in the OH931 *(left)* and NMFH-2 *(middle)* myxofibrosarcoma cell lines, and the western blots exhibited the expected bands using anti-MTAP. Two clones of *shMTAP* stably downregulated MTAP expression in NMFH-1 cells exhibiting decreased protein abundance *(right)*, compared with the non-targeting *shLacZ* controls. **(C)** The BrdU readout, indicating the level of proliferative activity, was significantly reduced in stable MTAP-transfected NMFH-2 cells *(left)*, but increased in MTAP-silenced NMFH-1 cells *(right)*, compared with the corresponding controls. Depending on the MTAP expression status, only the expression level of cyclin D1 in western blots was consistently altered as expected among the MTAP-reexpressing NMFH-2 cells and 2 MTAP-knockdown clones of the NMFH-1 cells. **(D)** Compared with the empty controls *(left)*, the MTAP-reexpressing NMFH-2 transfectants *(middle)* formed fewer colonies on soft agar, as expressed in the histogram *(bottom)*.

Because of physical proximity, *MTAP* inactivation by deletion in 9p21.3 was generally considered a bystander event concomitant with deletions of *CDKN2A/B* [13, 14]. Until recently, scholars have started noticing the tumor-suppressive attributes of *MTAP* that may independently inhibit carcinogenesis [9, 11]. We first characterized myxofibrosarcoma cell lines regarding their *MTAP* gene status and endogenous protein expression. Consistent with the aCGH findings, homozygous deletion in OH931 and NMFH-2 cells was confirmed using real-time PCR quantification without a detectable *MTAP* DNA copy (Figures 3A, S2A), reflective of the absence of MTAP protein expression. Endogenously expressing MTAP, the NMFH-1 cells were neither homozygously deleted at the *MTAP* gene nor methylated at its promoter (Figure 3A, S1A). Therefore, we applied *MATP*-bearing vectors and *shMTAP* to decipher how inactivated *MTAP* modulates myxofibrosarcoma phenotypes regarding various cancer hallmarks. The stably transfected and knockdown clones were validated using western blots (Figure 3B). In the NMFH-1 cells, the silencing of the *MTAP* transcript with 2 stable clones of *shMTAP* was determined using real-time quantitation (Figure S2B).

### MTAP expression inhibited cell proliferation and colony formation of myxofibrosarcoma primarily through the downregulation of cyclin D1

To elucidate the potential MTAP-associated antitumor function, we compared the BrdU readout of myxofibrosarcoma cells engineered to manifest various MTAP expression levels. In the MTAP-reexpressing NMFH-2 myxofibrosarcoma transfectants, the readout indicative of cell proliferation significantly reduced, whereas *shMTAP* effectively promoted cell proliferation in the NMFH-1 cells exhibiting endogenous MTAP (Figure 3C). By using an ECIS assay, we measured NMFH-2 cells for real-time cell proliferation, revealing a significantly slow cell growth rate in MTAP-reexpressing transfectants (Figure S2C). These observations supported that MTAP may dampen the proliferation of myxofibrosarcoma, prompting investigating whether MTAP expression modulates cell cycle regulators and inhibits colony formation. We performed western blotting to evaluate alterations in the G1- and G1/S-associated cyclins and cyclin-dependent kinases. Among these, only cyclin D1 expression was consistently downregulated in both MTAP-reexpressing NMFH-2 cells and *shLac*-transfected NMFH-1 cells (Figure 3C). However, the trend in alterations in cyclin E and CDK2 expression varied between exogenous expression and RNA interference approaches as well as between 2 *shMTAP* clones. In the flow cytometric analysis, MTAP-reexpressing NMFH-2 cells exhibited significant cell cycle arrest in the G1 phase and concomitant low percentages of cells in the S and G2 phases (Figure S2D). In a soft-agar assay, significantly small and few cell colonies were observed in MTAP-reexpressing NMFH-2 transfectants, substantiating the inhibition of MTAP in anchorage-independent growth (Figure 3D). These findings imply that cyclin D1 represents the predominant cell cycle regulator associated with the antiproliferative role of MTAP.

### MTAP expression inhibited cell migration and invasion

The results of wound-healing assays indicated significantly rapid wound closure in both NMFH-2 cells transfected with an empty vector and NMFH-1 cells silenced against endogenous MTAP (Figure S3A,C). According to matrigel invasion assays, the 2 conditions also yielded significantly increased invading tumor cells (Figure S3B), indicating enhanced metastatic propensity by MTAP deficiency in myxofibrosarcomas.

### MTAP expression conferred antiangiogenic function

**Figure 4:**
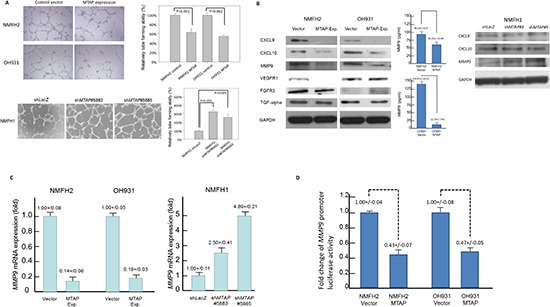
MTAP governs tumor angiogenesis by the transcriptional repression of MMP-9 **(A)** The well-formed HUVEC tubes decrease when exposed to conditioned media from MTAP-reexpressing transfectants of NMFH-2 and OH931 cells. Conversely, MTAP knockdown promoted angiogenesis in both *shMTAP*-transduced clones of the NMFH-1 cells *(left)*. The relative level of angiogenic tube-forming activity is expressed in the histograms *(right)*. **(B)** Of the differentially expressed genes identified from the RT-PCR expression array, western blotting validation for both MTAP-reexpressing transfectants exhibited correspondingly decreased protein expression of CXCL-9, CXCL-10, and MMP-9 *(left)*. The MMP-9 protein concentration secreted into the conditioned media was significantly reduced in MTAP-reexpressing NMFH-2 and OH931 cell lines *(middle)*. Only the level of MMP-9 protein expression was significantly upregulated in the cross-validation of MTAP-silenced NMFH-1 cells *(right)*. **(C)** As a representative potential downstream mediator of MTAP, the *MMP-9* mRNA expression levels were significantly downregulated in the MTAP-reexpressing NMFH-2 and OH931 cells *(left)* but elevated by MTAP knockdown using quantitative RT-PCR *(right)*. **(D)** Luciferase activity of *MMP-9* promoter construct was significantly lower in the NMFH-2 and OH931 cells transfected with the MTAP-expressing vector than in the empty controls. All experiments were performed in triplicate, and the results are expressed as mean ± SD.

Recently, we reported that aberrant loss of argininosuccinate synthetase (ASS1) caused by promoter methylation may contribute to the enhanced angiogenesis in myxofibrosarcomas [15]. Because MTAP is linked to polyamine metabolism and adenine and methionine salvage [16, 17], examining whether *MTAP* inactivation promotes angiogenesis by perturbing the metabolic balance of sarcoma cells is conceivable, similar to the scenario involved in deregulated ASS1. Under various experimental conditions used to manipulate MTAP expression, 3 myxofibrosarcoma cell lines were assessed for the formation of HUVEC tubes incubated with corresponding conditioned media. Using HUVEC assays, the angiogenic capability significantly decreased to 55% and 60% in the MTAP-reexpressing OH931 and NMFH-2 transfectants, respectively, but significantly increased up to 2~3-fold in the MTAP-silenced NMFH-1 cells (Figure 4A). Because of increased angiogenesis in the MTAP-deficient state, we used an RT-PCR expression array to explore the potential mediators relevant to MTAP-regulated angiogenesis. Lacking upregulated genes in this angiogenesis-associated panel, MTAP-reexpressing NMFH-2 cells showed significantly reduced mRNA expression of 6 candidates (Table S5). Of these, CXCL-9, CXCL-10, and MMP-9 proteins were concomitantly downregulated using western blots in both NMFH-2 and OH931 MTAP-reexpressing cell lines, the conditioned media of which also consistently demonstrated decreased MMP-9 protein concentration using ELISA (Figure 4B). Real-time RT-PCR showed decreased expression of *MMP-9* mRNA in both MTAP-reexpressing myxofibrosarcoma cell lines (Figure 4C). In the MTAP-silenced NMFH-1 cells used for cross-validation, MMP-9 was the only molecule that exhibited upregulated mRNA and protein expression (Figure 4B, C). The luciferase activity of *MMP-9* promoter significantly reduced in the MTAP-reexpressing OH931 and NMFH-2 cells (Figure 4D). These data demonstrated that MTAP deficiency enhanced the transcriptional upregulation of *MMP-9* to promote angiogenesis in myxofibrosarcomas.

### MTAP reexpression inhibited growth of xenografted tumors

**Figure 5:**
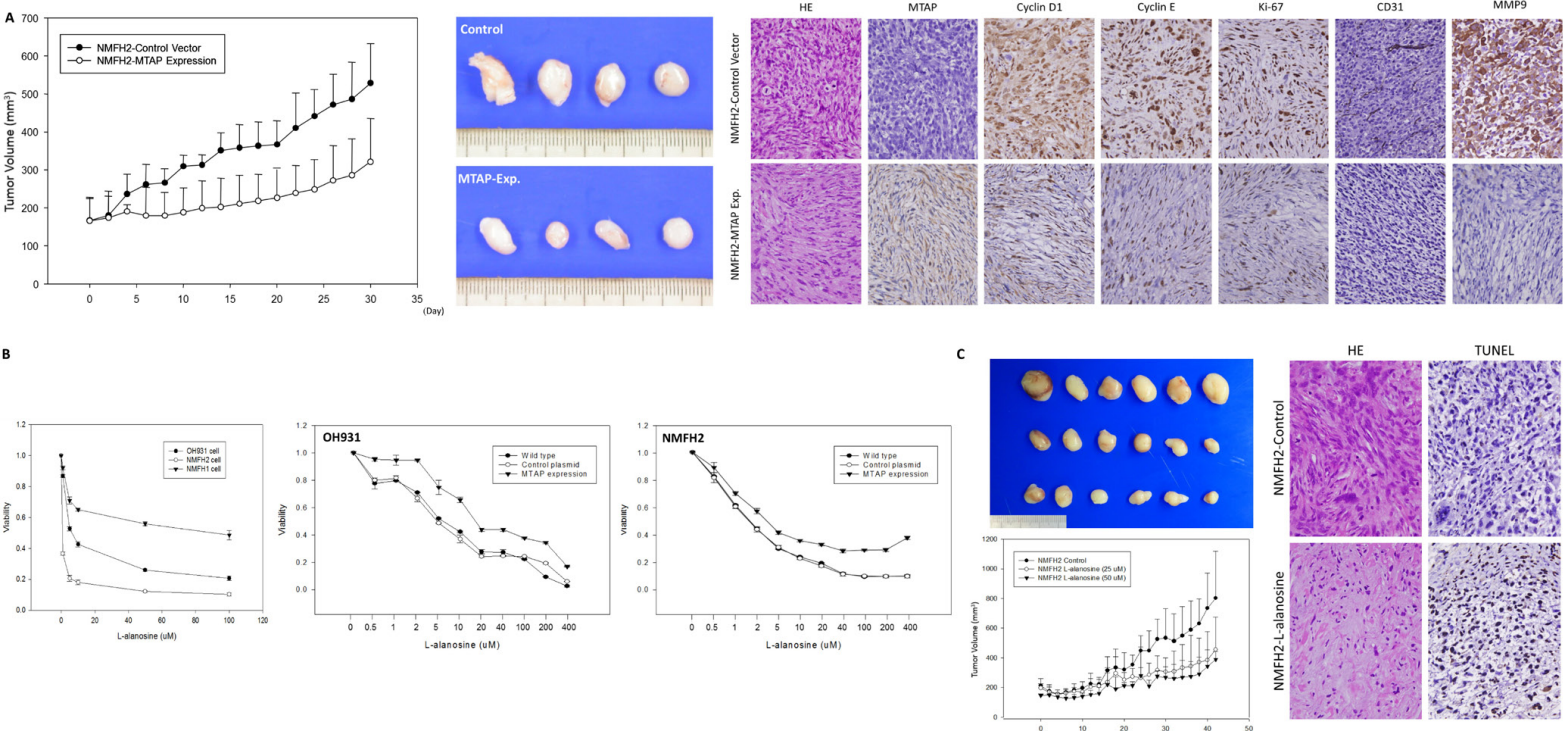
*In vivo* tumor suppressive function of MTAP and the inhibition of L-alanosine in MTAP-deficient myxofibrosarcoma **(A)** The average tumor volume of the empty vector-transfected NMFH-2 xenografts was significantly larger than that of the MTAP-reexpressing counterparts *(left)*. On Day 30, the excised xenografts were grossly larger and heavier in the control group than in the MTAP-reexpressing group *(middle)*. Histologically, control xenografts (upper panel) comprise MTAP-deficient high-grade pleomorphic sarcoma cells that exhibit frequent mitotic figures, the increased expression of cyclin D1, cyclin E, Ki-67, and MMP-9, and an increased microvascular density based on CD31 staining. However, contrasting results were observed in the MTAP-reexpressing group (lower panel), which exhibited low-grade MTAP-expressing spindle cells *(right)*. **(B)** L-alanosine effectively attenuated the viability of MTAP-deficient NMFH-2 and OH931 myxofibrosarcoma cell lines, and MTAP-expressing NMFH-1 cells were relatively resistant to L-alanosine, demonstrating >50% viable sarcoma cells, even at 100 μM *(left)*. Unlike the sensitivity in the parent lines and empty controls, MTAP reexpression caused decreased susceptibility to L-alanosine in the OH931 *(middle)* and NMFH-2 *(right)* cells. **(C)** The average tumor volume was significantly larger in the PBS-treated group than in the groups treated with 25 μM and 50 μM of L-alanosine *(left)*. On Day 42, the excised xenografts remained macroscopically larger and heavier in the control group, exhibiting no dose-dependent difference between the groups treated with 25 μM and 50 μM of L-alanosine *(middle)*. In contrast to the high-grade pleomorphic histology in the control xenografts lacking apparent apoptosis, the L-alanosine-treated group exhibited increased numbers of fibrohyaline collagen fibers and TUNEL-labeled apoptotic cells *(right)*.

The *in vivo* inhibitory effect of MTAP on tumor growth was analyzed in NMFH-2 xenografts exhibiting ectopic MTAP reexpression versus empty controls (Figure 5A). The xenografts reexpressing MTAP exhibited a significantly small average tumor volume from Day 7 until sacrifice on Day 30, on which the excised specimens were evidently larger and heavier in the controls than were those in the MTAP-reexpressing group. The control xenografts exhibited more high-grade pleomorphic cells in a fibromyxoid matrix and a higher mitotic rate (*P* < 0.001) compared with the MTAP-reexpressing xenografts primarily composed of low-grade spindle cells. Immunohistochemically, MTAP-reexpressing xenografts exhibited a significantly reduced labeling of cyclin D1, cyclin E, and Ki-67, as well as decreased intratumoral vessels stained with the CD31 endothelial marker, substantiating the antiproliferative and antiangiogenic attributes of MTAP *in vivo*.

### MTAP protein deficiency was associated with increases of proliferative index, microvessel density, and MMP-9 expression level in primary myxofibrosarcomas

In a subset common cohort of 40 primary myxofibrosarcomas yielding informative scoring results (Table S6, Figure S4), we further corroborated the *in vitro* findings in clinical samples that MTAP protein deficiency was highly associated with increased Ki-67 labeling index (*p* < 0.001), microvessel density (*p* = 0.002) assessed by CD31 staining [18], and MMP-9 expression levels (*p* < 0.001) evaluated by H-score method [19].

### L-alanosine inhibited MTAP-deficient myxofibrosarcoma cells and xenografts by inducing apoptosis

Because L-alanosine can completely abrogate AMP supply in MTAP-deficient cells, we investigated whether L-alanosine can effectively inhibit this aggressive subset of myxofibrosarcomas by targeting the methionine de novo pathway [13]. All myxofibrosarcoma cell lines were incubated with indicated doses of L-alanosine for 72 h; this attenuated the cell viability in only the MTAP-deficient parent NMFH-2 and OH931 cells yielding IC50 values of approximately 1 μM and 5 μM, respectively, but not in the MTAP-expressing NMFH-1 cells (Figure 5B). Flow cytometry demonstrated a significantly increased sub-G1 population in both L-alanosine-treated OH931 and NMFH-2 cells, and the induction of apoptosis, particularly prominent in the NMFH-2 cells, was validated using annexin V/propidium iodide staining assays (Figure S5). Stable MTAP-reexpressing OH931 and NMFH-2 cells were employed to explore how MTAP expression affected the susceptibility of myxofibrosarcoma cells to L-alanosine (Figure 5B). This approach significantly reverted the attenuated cell viability caused by L-alanosine, indicating that its antitumor selectivity was attributable to the MTAP deficiency in myxofibrosarcoma cells.

We evaluated the *in vivo* efficacy of L-alanosine in NMFH-2 xenografts (Figure 5C). From Day 25 posttreatment until Day 42 at sacrifice, significantly smaller tumor volumes were observed in both groups treated with L-alanosine at 25 μM or 50 μM, compared with the PBS controls. However, no dose-dependent therapeutic effect was observed. The control xenografts exhibited high-grade pleomorphic histology characterized by frequent atypical mitoses, whereas the L-alanosine-treated specimens exhibited quiescent or apoptotic-appearing cells in a fibromyxoid backdrop containing increased hyaline collagenous fibers. In the L-alanosine-treated xenografts, significantly increased TUNEL-labeled apoptotic cells were observed (*P* < 0.001), substantiating the *in vivo* apoptosis-inducing effect of L-alanosine.

## DISCUSSION

Myxofibrosarcoma is a common, and genetically complex sarcoma of histological and biological heterogeneity, and its therapeutic mainstay is conducting radical surgery to achieve clear margins [2, 3]. The lack of effective molecular markers and standardized chemoradiotherapeutic regimen(s) dictates an unfavorable prognosis in primary advanced and recurrent myxofibrosarcomas [2, 3]. By integrating various methodologies, we identified the clinical, biological, and therapeutic relevance of MTAP deficiency, which was observed in 37% of primary myxofibrosarcomas, strongly linked to homozygous deletion or promoter methylation, and independently predictive of adverse survival. However, no inactivating mechanism was detected in the 3 MTAP-deficient cases, suggesting that MTAP downregulation was caused by alternative molecular defects such as small deletions of 5′ regions [20, 21]. On the other hand, homozygous deletion of *MTAP* gene was detected in four cases interpreted as MTAP-expressing. This conflicting finding was most likely attributable to the existence of intratumoral heterogeneity. Whatever whole-block or tissue microarray sections, the MTAP-positive tumor cells only ranged from 10% to 20% at most, which might not be isolated in the process of laser capture microdissection and therefore result in an underestimated gene ratio skewed toward homozygous deletion. Compared with the equally indolent *MTAP*-hypermethylated and MTAP-expressing myxofibrosarcomas, *MTAP-*homozygously deleted tumors were often high-grade and clinically aggressive. This variation in the prognostic effect of *MTAP* gene abrogation between distinct mechanisms in myxofibrosarcoma progression was concordant with our previous findings regarding GISTs [22], indicating the probable random occurrence of *MTAP* promoter methylation in the early phase of myxofibrosarcoma tumorigenesis. However, *MTAP* homozygous deletion represents a relatively late genetic event in the tumor progression, which may confer survival advantage to aggressive subclones of myxofibrosarcoma cells and thereby culminate in negative prognostic impact.

MTAP depletion is not uncommon in human malignancies and is primarily attributable to homozygous deletions spanning 9p21.3. MTAP deficiency may occur without the loss of adjoining *CDKN2A* in non-small cell lung cancers and gliomas [13, 14, 23–26]. Instead of genomic losses, promoter hypermethylation represents the preponderant inactivating mechanism in MTAP-deficient hepatocellular carcinomas [21]. Functionally, MTAP reexpression in *MTAP*-deleted breast cancer cells may inhibit anchorage-independent growth and *in vivo* tumorigenicity [9]. Although mice heterozygous for germline *MTAP* mutations did not fully recapitulate the genetic events in clinical specimens, they were prone to developing T-cell lymphomas; however, homozygous inactivation was embryonically lethal in the MTAP-knockout murine model [11]. Because of these conflicting findings, whether the loss of MTAP activity is pathogenically relevant remains debated [9, 11, 12]. Given the clinical relevance of MTAP deficiency in myxofibrosarcomas, we characterized the biology of MTAP expression to clarify its functional effects on cancer hallmark-related phenotypes. The antiproliferative function of MTAP by the downregulation of cyclin D1 was validated using both MTAP knockdown in MTAP-expressing NMFH-1 cells and MTAP-reexpression in MTAP-deficient NMFH-2 cells. Regarding the latter condition, the growth-inhibiting attribute of MTAP was reinforced by the reduced proliferation evidenced in the ECIS measurements, few colonies in the soft-agar assays, and low-grade histology of the corresponding xenografted specimens exhibiting an underexpression of Ki-67 and cyclin D1. We also corroborated the suppressive role of MTAP in metastasis, which is the leading cause of sarcoma-associated mortality and critically involves the regulation of cell motility and transgression through an extracellular matrix [2, 27, 28]. In MTAP-reexpressing NMFH-2 cells, delayed closure in wound-healing assays and decreased invading cells through matrix-coated wells were measured and cross-validated by the reverse results of MTAP-silenced NMFH-1 cells. Clinically, the independent effect of MTAP deficiency on adverse MFS in myxofibrosarcomas was reflective of these MTAP-associated antimigratory and antiinvasive functions.

In MTAP-reexpressing and *shLacZ*-transduced myxofibrosarcoma cells, angiogenic capability was significantly impaired, as indicated by reduced levels of HUVEC-derived tube formation. Collectively, these findings corroborated endogenous MTAP as a functional suppressor that governs myxofibrosarcoma pathogenesis by inhibiting tumor proliferation, metastasis, and angiogenesis. In the luciferase reporter assay, the antiangiogenic effect of MTAP was determined to primarily link to the transcriptional repression of *MMP-9*, ultimately attenuating mRNA and protein expression in the myxofibrosarcoma cells. In the MTAP-deficient xenografts and myxofibrosarcoma specimens, MMP-9 overexpression was present in the tumor cells and extracellular stroma and associated with increased microvessel density. These exemplified the oncogenic role of MMP-9 in myxofibrosarcoma pathogenesis and corresponded with previous reports on its associations with aggressiveness in various cancer types [15, 28]. Kiroviski reported that MTAP knockdown upregulated MMP-9 expression through the accumulation of 5′-deoxy-5′-methylthioadenosine in hepatocellular carcinomas [29]. However, they neither elucidated the mechanistic link between MTAP deficiency and MMP-9 nor characterized the functional effect of this perturbed regulatory chain on angiogenesis and metastasis.

Because MTAP is not a transcriptional factor, it is conceivable to hypothesize that MTAP deficiency indirectly transactivates an *MMP-9* promoter, causing resultant aggressive phenotypes. Recently, we reported that the antiangiogenic function of ASS1, a rate-limiting enzyme converting citrulline into arginine in the urea cycle, was also attributable to the inhibition of ASS1 on MMP-9 expression in myxofibrosarcomas [15]. Evidently, aberrant losses of both MTAP in methionine salvage and ASS1 in arginine metabolism may upregulate ornithine decarboxylase (ODC) and subsequently induce overproduction of putrescine and other polyamines [17, 30, 31]. This alteration is one of the first processes involved in proliferating cells preceding nucleic acid and protein syntheses and recently has been reported to promote tumor development and progression involving multiple cancer hallmarks [16, 30]. For instance, ODC transformants were reported to increase secreted MMP-2 *in vitro* to enhance matrix degradation [30]. Analogously, MMP-9 overexpression in the ASS1-deficient and MTAP-deficient myxofibrosarcomas is likely mediated by overexpressed ODC, which converges growth-promoting signals to orchestrate transcriptional regulation.

The disabled methionine salvage in *MTAP*-inactivated myxofibrosarcomas renders this aggressive tumor subset a tumor-specific metabolic feature for use in L-alanosine therapy, which targets adenylosuccinate synthetase to abolish de novo AMP synthesis from IMP [13, 14, 30]. *In vitro* and *in vivo*, the growth inhibition of MTAP-deficient tumors by L-alanosine was partially ascribed to the induction of apoptosis, which was evidenced by increased Annexin-V and TUNEL labeling in myxofibrosarcomas and reported to be mitochondria-dependent in mantle cell lymphomas [24]. In addition, we demonstrated that myxofibrosarcomas fundamentally depended on MTAP deficiency regarding their susceptibility to L-alanosine, which effectively inhibited the MTAP-deficient cell lines and derived xenografts and was well-tolerated by the mice. Since several antimetabolites targeting purine synthesis are available and additional drugs are in development [32–34], the findings of this study suggest that MTAP-deficient myxofibrosarcomas may benefit from the chemoselective targeting of the defects in methionine-adenine recycling and AMP synthesis.

In summary, MTAP deficiency in myxofibrosarcomas, and particularly that caused by homozygous deletion, negatively affects prognosis. *In vitro* and *in vivo*, MTAP may act as a tumor suppressor, serving antiangiogenic, antiproliferative, and antimigratory or antiinvasive functions, and its deficiency represents a potential therapeutic target of myxofibrosarcomas.

## MATERIALS AND METHODS

### Analysis of aCGH

Twelve fresh tumor specimens and 3 myxofibrosarcoma cell lines (OH931, NMFH-1, and NMFH-2) underwent oligonucleotide-based aCGH profiling (NimbleGen), as previously detailed [7, 15]. The raw data from the aCGH profiling were log2-transformed and output into Nexus software (BioDiscovery). To delineate the breakpoints in the genome-wide array probes, the gains and losses in the critical regions of the CNAs were defined as log2 ratios of ≥ +0.20 and ≤ −0.20, respectively. The amplification and homozygous deletion were defined as log2 ratios of ≥ +0.50 or ≤ −0.50, respectively. To unravel causal genes exhibiting copy number-driven deregulated expression, the common regions of alteration were filtered to identify consecutive makers, and their proportions were ^ 20% in the tumor and cell line samples analyzed.

### Tumor materials

An institutional review board (97-2190A3) approved the retrieval of myxofibrosarcomas resected with curative intent. Those undergoing neoadjuvant radiation, chemotherapy or aCGH profiling were excluded. Table 1 lists the 87 independent primary formalin-fixed myxofibrosarcomas successfully analyzed for *MTAP* gene dosage, promoter methylation, and immunoexpression.

### Immunohistochemistry

Tissue microarray sections were antigen-retrieved, incubated with antibodies targeting MTAP (1:100, Proteintech), Ki-67 (1:200, abcam), MMP-9 (1:50, Epitomics), and CD31 (1:50, BD Pharmingen), and detected using the ChemMate EnVision kit. The cutoff value of < 10% cytoplasmic reactivity to define aberrant MTAP loss was previously described [22]. The scoring criteria of determining the levels of intratumoral microvessel density [18] and MMP-9 expression by H-score method [19] were as previously reported. Whole sections of xenografted specimens were stained with MTAP (1:50, Proteintech), cyclin D1 (1:100, Epitomics), cyclin E (1:20, Abcam), Ki-67 (1:200, abcam), MMP-9 (1:50, Epitomics), CD31 (1:50, BD Pharmingen), and TUNEL (Roche) for labeling apoptotic cells.

### *MTAP* dosage quantification

As described in Supplementary Method-S1, real-time polymerase chain reaction (PCR) was performed on *MTAP* exon 8 in duplicate for each case to detect the *MTAP* gene dosage. *PIK3R1* (5q13.1) was the reference gene that exhibited no detectable CNAs in the aCGH. Standard curves were constructed using serial dilutions of human genomic DNA (Clontech). For each specimen, approximately 3000 tumor cells were microdissected using the Veritas LCM machine (Arcturus) to extract genomic DNA. The *MTAP*/*PIK3R1* ratio < 0.2 was considered a homozygous deletion, assuming that the normal tissue contamination and intratumoral heterogeneity accounted for < 20% of the experimental deviation. Regarding the 3 myxofibrosarcoma cell lines, homozygous deletion was determined when no *MTAP* gene copy was detected but *PIK3R1* was amplifiable, i.e. the *MTAP*/*PIK3R1* ratio approximating zero.

### Methylation-specific PCR

Genomic DNA was extracted from formalin-fixed tissue, bisulfite-converted, and PCR-amplified with primers targeting methylated and unmethylated promoters of *MTAP* gene, as previously described [22].

### Cell culture, transfection, and RNA interference

The culture conditions of the OH931, NMFH-1, and NMFH-2 myxofibrosarcoma cell lines, CCD966SK dermal fibroblasts, and human umbilical venous endothelial cells (HUVECs, H-UV001) were previously described [15] and were authenticated using short tandem-repeat fingerprinting in December, 2013.

Regarding the MTAP reexpression in *MTAP*-deleted OH931 and NMFH-2 cells, a full-length pMTAP-DDK-Myc expression plasmid and empty pCMV6 vector were purchased from Origene and validated using sequencing. In a 24-well plate, 105 cells from OH931 or NMFH-2 myxofibrosarcoma cell lines were seeded and incubated with lipofectamine 2000 (Invitrogene) for 4 h at 37°C to transfect various plasmids (1.5 μg), then cultured for a further 24 h at 37°C, and lysed with Passive Lysis Buffer (Promega). Cells stably expressing MTAP or DDK-Myc tags alone were selected using neomycin. The transfected cells were analyzed for exogenous MTAP expression using western blotting. To evaluate the *MMP-9* promoter activity, an empty pGL vector and pG-*MMP-9*-promoter were cotransfected with a pCMV6 control or a pMTAP-DDK-Myc vector into myxofibrosarcoma cells [35]. After 48 h, the cells underwent luciferase assays by using a dual luciferase assay kit (Promega) and normalized for the transfection efficiency of pRL vector.

To silence the endogenous MTAP expression in NMFH-1 cells, lentiviral vectors were obtained from the Taiwan National RNAi Core Facility. Viruses were produced by transfecting HEK293 cells with the control pLKO.1-*shLacZ* (TRCN0000072223: 5′-TGTTCGCATTAT CCGAACCAT-3′) and pLKO.1-*shMTAP* (TRCN0000005883: 5′-CCTGAATGATTTAGACAAC-3′ and TRCN0000005885: 5′-CCTTCTATGATGGAAGTCA-3′) using Lipofectamine 2000. For viral infection, 3 × 106 NMFH-1 cells were incubated with 8 mL of lentivirus in the presence of polybrene, followed by puromycin selection to produce stable clones of lentivirus-transduced cells.

### Quantification of transcripts of MTAP and angiogenesis-associated genes

As detailed in Method-S2, real-time reverse-transcription PCR (RT-PCR) was performed using the ABI StepOnePlus™ System to measure *MTAP* mRNA abundance in all myxofibrosarcoma cell lines. To explore antiangiogenic mediators of MTAP, an RT-PCR expression array (PAHS-024, SABioscience) was profiled for NMFH-2 cells. Those genes differentially expressed between MTAP-reexpressing and empty conditions were identified based on *P* < 0.001, > 5-fold changes, and *Ct* values % 30 for both tested and housekeeping genes. These candidates were further screened in all myxofibrosarcoma cell lines using western blots to assay molecules exhibiting corresponding protein alterations under varying MTAP reexpression or knockdown experiments.

### Western blots

To visualize protein expression alterations, Method-S3 details the western blots, sources of primary antibodies against MTAP, G1- and G1/S transition-associated cell cycle regulators, and RT-PCR expression array-selected angiogenesis-associated mediators. The proteins consistently downregulated or upregulated by MTAP reexpression in MTAP-deficient OH931 and NMFH-2 cells were reappraised in endogenously MTAP-expressing NMFH-1 cells transduced with *shMTAP* or *shLacZ*.

### Enzyme-linked immunosorbent assay (ELISA)

After the aforementioned cross-validations were conducted, the identified molecule(s) exhibiting the same trend in changes in protein abundance, as modulated by the MTAP expression level, underwent ELISA to quantify their concentration in corresponding conditioned media, as detailed in Method-S4.

### Pharmacological assessment

Parent OH931, NMFH-2, and NMFH-1 cells, as well as the MTAP- or empty vector-transfected OH931 and NMFH-2 cells, were treated with a vehicle control using 0.9% phosphate-buffered saline (PBS) or L-alanosine (Santa Cruz Biotech) at indicated doses for 72 h.

### Functional assays

To elucidate the functional alterations associated with MTAP reexpression, MTAP knockdown, and L-alanosine treatment, various cancer phenotypes were evaluated using cell viability, bromodeoxyuridine (BrdU), electric cell-substrate impedance sensing (ECIS), cell cycle kinetics, soft-agar, HUVEC tube formation, and wound-healing, invasion, and apoptosis assays in Methods-S5-S11.

### Animal xenografts

The animal use committee approved the protocol 103101401. To elucidate the *in vivo* function of MTAP reexpression, 4 × 106 NMFH-2 myxofibrosarcoma cells transfected with the MTAP-expressing plasmid or empty vector (*n* = 8 each) were inoculated into the flanks of 16 severe combined immunodeficient (SCID) mice (BioLasco) and allowed to grow until 30 d after cell implantation. To analyze the *in vivo* therapeutic effect of L-alanosine, 4 × 106 parent NMFH-2 cells were inoculated into 24 SCID mice, which were allowed to grow until 10 d postinjection (Day 0), and then randomized into 3 groups (*n* = 8 each) receiving 25 μM or 50 μM of L-alanosine, or PBS. The treatment was continued for 6 wk and the mice were euthanized 42 d posttreatment. The tumor volume was calculated using the formula: V=π/6 × length (mm) × width (mm)2.

### Statistical analysis

The associations and comparisons among various parameters were evaluated using chi-square, Student *t*, or Mann-Whitney U test as appropriate to assess the *MTAP* gene status and MTAP immunoexpression. Follow-up data were available for 83 patients, and the median follow-up time was 30.6 mo. The endpoints were metastasis-free survival (MFS) and disease-specific survival (DSS). Univariate survival analyses were compared using a log-rank test. A multivariate model was performed using a Cox regression, including parameters with univariate *P* < 0.05. For cell line and xenograft samples, a Student's *t*-test was used to analyze the functional and pharmacological assays.

## SUPPLEMENTARY METHODS, TABLES AND FIGURES


