# Supplementary material for: Downregulated MTAP expression in myxofibrosarcoma: A characterization of inactivating mechanisms, tumor suppressive function, and therapeutic relevance
Source: Oncotarget. 2014 Oct 24;5(22):11428–41. doi: 10.18632/oncotarget.2552 (PMC4294342; doi:10.18632/oncotarget.2552)
Supplement: Supplementary file 1 [file oncotarget-05-11428-s001.pdf]

SUPPLEMENTARY METHODS, TABLES AND FIGURES

Method S1: Conditions of real-time quantitative PCR used to measure MTAP gene dosage

The DNA extracted from myxofibrosarcoma cell lines and LCM-isolated pure myxofibrosarcoma cells was quantified using an ND-1000 spectrophotometer (Nanodrop). The relative fold change in the *MTAP* gene copy number between normal and tumor tissues or various cell lines was measured using a quantitative PCR assay, incorporating the comparative threshold cycle (C_t) method. The TaqMan assays targeting *PIK3R1* (Hs06028467_cn) in 5q13.1 and exon 8 of the *MTAP* gene (Hs02079487_cn) in 9p21.3 were ordered from Applied Biosystems and separately amplified using the ABI StepOnePlus™ Real-Time PCR System (Applied Biosystems). The PCR was performed in a total volume of 20 µL in each well, which contained 10 µL of TaqMan Genotyping MasterMix, 20 ng of genomic DNA, and 1 µL of 20x TaqMan assay probe. The PCR conditions involved an initial denaturation step of 95°C for 10 min, followed by 40 cycles at 95°C for 15 s, and 60°C for 1 min. All reactions were conducted in duplicate, and a negative control with no template was run in parallel with each PCR reaction.

The *MTAP* gene copy numbers of the tumor samples were derived by calculating the gene ratios of *MTAP* to *PIK3R1* by using the $2^{\Delta\Delta Ct}$ method and normalizing them to the gene copy number of *PIK3R1*, defined as 2. A reference standard curve was established using input templates of 100, 20, 8, and 0.4 ng human genomic DNA (Clontech) as the calibrator. This gene ratio (*R*) was determined using the following formula: $R = 2^{\Delta\Delta Ct}$, where $\Delta\Delta Ct = \Delta Cp$ (calibrator) - ΔCp(sample) and ΔCp = Cp(*MTAP*) - Cp(*PIK3R1*). The average ΔCp of the standard curve points was set as ΔCp(calibrator) at a gene ratio of 1. Accordingly, a normalized 2-gene copy test sample was expected to yield a gene ratio of 1.

Regarding the formalin-fixed samples, the *MTAP*/*PIK3R1* gene ratio < 0.2 was considered as representing homozygous deletion based on the assumption that the normal tissue contamination and intratumoral heterogeneity should collectively account for <20% of experimental deviation. The samples exhibiting measured gene ratios between ≧ 0.2 and ≤ 0.8, between > 0.8 and ≤1.2, and > 1.2 were classified as the hemizygous deletion, normal copy number, and gain of the *MTAP* gene, respectively. Regarding the 3 myxofibrosarcoma cell lines, homozygous deletion was determined when the gene ratio approximated 0 and no *MTAP* gene copy was detectable in the presence of amplifiable *PIK3R1*.

Method S2: Real-time RT-PCR used to quantify MTAP transcript and to identify its candidate angiogenesis-regulating mediators by using an RT-PCR array

The RNeasy Mini kit (Qiagen, Valencia, CA, USA) was used to extract total RNA from the NMFH-1 myxofibrosarcoma cell lines transduced with stable *shMTAP*-mediated knockdown of endogenous MTAP expression and *shLacZ* controls. The RNAs were further reverse-transcribed using the SuperScript™ III First-Strand Synthesis System (Invitrogen, Carlsbad, CA, USA) according to the manufacturer instructions. Real-time PCR array was performed to quantify the expression level of *MTAP* transcript by using predesigned TaqMan assay reagents (*MTAP* Hs00559618_mL, *MMP-9* Hs00234579_mL, and POLR2A [i.e., RNA polymerase polypeptide A] Hs01108291_mL, from Applied Biosystems, Foster City, CA, USA) and the ABI StepOnePlus™ Real-Time PCR System. The obtained data were normalized based on the expression of the *POLR2A* housekeeping transcript. The relative expression fold of the *MTAP* or *MMP-9* transcript was then determined based on $2^{-\Delta\Delta Cp}$, where $\Delta\Delta C_T = \Delta C_T$ () - $\Delta C_{T\text{ (sarcoma cells)}}$ and $\Delta C_{T\text{ (CCD966SK fibroblasts)}}$ represented the C_T of MTAP subtracted from the C_T of *POLR2A*. Only samples yielding a C_T value < 32 for *POLR2A* were considered to exhibit acceptable RNA quality and were included in the analyses.

To explore potential mediators of MTAP that could implicate its antiangiogenic function, we applied an RT-PCR array (PAHS-128, SABioscience) to analyze the differential expression profiles of *MTAP*-null OH931 and NMFH-2 cells stably engineered to reexpress MTAP and of MTAP-expressing NMFH-1 cells stably silenced by *shMTAP*. The PCR reactions for 84 angiogenesis-associated genes were run on the ABI StepOnePlus™ System (Applied Biosystems). Expression was normalized to housekeeping genes and presented as fold expression relative to the empty vector or *shLacZ* controls.

Method-S3: Western blotting

Cell lysates containing 25 µg of protein were separated using 4%–12% gradient SDS-PAGE, transferred onto PVDF membranes (Amersham), and probed using antibodies against MTAP

(Proteintech, 1:500), cyclin D1 (Santa Cruz, 1:200), cyclin E (Santa Cruz, 1:200), CDK2 (Santa Cruz, 1:500), CDK4 (Santa Cruz, 1:200), CXCL9 (Novus Biologicals, 1:1000), CXCL10 (Epitomics, 1:1000), MMP-9 (Epitomics, 1:100), VEGFR1 (Epitomics, 1:1000), FGFR3 (Epitomics, 1:500), and TGF-α (Abcam, 1:200). GAPDH (Chemicon, 1:3000) was used as the loading control. After incubation with the secondary antibody, the proteins were visualized using the enhanced chemiluminescence system (Amersham).

Method S4: Enzyme-linked immunosorbent assay used to quantify the protein concentration in conditioned media

MTAP-reexpressing myxofibrosarcoma cells and vector-alone controls were plated in a 24-well culture dish (5×10^5/mL) and maintained for 96 h in serum-free media. After being filtered, the supernatant of media from various transfectants was percolated through a 0.22 um filter to measure the concentration of MMP-9 by using ELISA (R&D). The absorbance of the samples was determined using an ELISA reader (Promega) at 450 nm, using the absorbance at 560 nm as a reference.

Method-S5: Cell viability assay

The investigated cells were plated on gelatinized 96-well plates at a density of 5×10^3 cells per well and analyzed on an ELISA microplate reader at 492 nm by using the XTT assay kit (Roche).

Method-S6: Bromodeoxyuridine (BrdU) assay and electric cell-substrate impedance (ECIS) sensing assays

DNA synthesis was assessed using ELISA-based and colorimetric BrdU assays (Roche Diagnostics). Various plasmid vector-transfected, *shRNA*-transduced, and L-alanosine- or PBS-treated cells were plated onto a 96-well plate at a density of 2000, 3000, and 3000 cells per well for OH931, NMFH-1, and NMFH-2, respectively. In addition, DNA syntheses were evaluated at 24, 48, and 72 h. After incubation with BrdU for 3 h at 37°C in 5% CO_2, the labeling medium was removed, and the samples were fixated, then underwent a final incubation with an anti-BrdU-POD solution. The absorbance of the samples was measured using an ELISA reader (Promega) at 450 nm, and the absorbance at 690 nm was used as reference.

To measure cell proliferation accurately, an 8-electrode culture dish (Applied Biophysics) involving one electrode per well was precoated with 400 uL of media and experimental additives and then incubated for 2 h. A total of 4×10^4 MTAP-reexpressing or empty control NMFH-2 cells were then seeded into the well. The ECIS collected the resistance values every 10 min for a total run time of 72 h.

Method-S7: Flow cytometric analysis of cell cycle

Stable pools of NMFH-2 myxofibrosarcoma cells transfected with MTAP-expressing plasmids or empty vectors were pelleted and fixed overnight in 75% cold ethanol at −20°C. The cells were washed twice in cold PBS containing 10 mg/mL of DNase-free RNase. Subsequently, the cells were labeled using propidium iodide (PI) at a concentration of 0.05 mg/mL and analyzed using the FACScan flow cytometer (BD Biosciences) on WinMDI2.9 software to determine the percentage of cells in each phase of the cell cycle. In all experiments, more than 10^4 cells were sorted after gating the fixation artifacts and cell debris.

Method-S8: Soft agar assay

To assess the level of anchorage-independent growth, 2×10^4 NMFH-2 myxofibrosarcoma cells transfected with pCMV6-ASS1-DDK-Myc or empty vectors were trypsinized, mixed in 1 mL of 0.3% agar in a complete medium, and then seeded onto a 6-well plate containing 1.5 mL of 0.6% agar in a complete medium. After being further cultured for 14 d, the colonies were visualized using 0.5% P-iodonitrotetrazolium violet stain overnight and those >100 μm were counted under the microscope in 10 random fields in 3 independent triplicate assays.

Method-S9: Wound healing and invasion assays

An artificial “wound” was created using a 200 μL pipette tip on confluent cell monolayers on 6-well plates. Photographs were captured at 0, 12, 24, 48, and 72 h. Quantitative analysis of the wound closure was calculated by measuring the percentage of healed area relative to the initial wound at Day 0.

The level of cell invasion was determined using a 24-well Collagen-based Cell Invasion Assay (Millipore). Briefly, each insert was added to a 0.3-mL serum-free medium to rehydrate the collagens, replaced with 0.3 mL of a serum-free suspension of 10^6 cells in the upper chamber, and incubated for 24 h to enable the cells to migrate toward the lower chamber containing 10% fetal bovine serum. After noninvading cells in the upper chamber were removed, cells invading through the inserts were stained using provided dye, dissolved in an extraction buffer, and transferred to 96-well plates for colorimetric reading at 560 nm.

Method S10: HUVEC tube formation assay

To analyze the effect of MTAP on tumor cell-induced tube formation in HUVECs, the conditioned media of myxofibrosarcoma cells exhibiting exogenous MTAP reexpression, *shMTAP*, or their corresponding controls, were harvested using serum-free starvation for 96 h. The 96-well plates were precoated with matrigel after polymerization of the matrix at 37°C. The HUVECs were then plated at a density of 2×10^4 cells/well and incubated using various conditioned media. After 6 h of treatment, the capillary-like tube formation was assessed by measuring the length of the capillary mesh under an inverted photomicroscope.

Method-S11: Cell apoptosis analysis

For the cell apoptosis analysis, 10^5 NMFH-2 or OH931 cells/Petri dish (60 mm) were plated for 24 h, harvested after incubation with L-alanosine or vehicle controls for the indicated doses and time, and then stained using the Annexin V-FITC kit (Bender MedSystems) containing propodium iodine for 15 min. The cell percentages at the stages of early apoptosis and late apoptosis were calculated based on 3 independent experiments.

Supplementary Table S1. The regions on chromosome 9 recurrently showing copy number alterations in at least 20% of tumor and cell line samples analyzed by aCGH. In the short arm of chromosome 9, short stretches of DNA gains are interspersed among 5 major discontinuous deletion cores. Of these, 9p22.2 - p21.1 harbors the 9p21.3 region which are differentially lost in cases exhibiting clinical aggressiveness. In contrast, the copy number alterations in the long arm of chromosome 9 are predominantly DNA gains, except for the telomeric part where the 9q34 region shows DNA losses.

Region	Region Length	Cytoband Location	Event	Genes
chr9:0–9, 584, 460	9584460	p24.3 - p23	CN Loss	67
chr9:11, 759, 609–11, 996, 968	237359	p23	CN Gain	0
chr9:10,203,247-17,168,886	6965639	p23 - p22.2	CN Loss	28
chr9:17,537,683-32,074,169	14536486	p22.2 - p21.1	CN Loss	84
chr9:33,409,491-33,959,515	550024	p13.3	CN Gain	19
chr9:34,259,404-34,528,298	268894	p13.3	CN Gain	8
chr9:32,771,948-37,112,632	4340684	p21.1 - p13.2	CN Loss	134
chr9:35,197,017-35,612,583	415566	p13.3	CN Gain	11
chr9:37,459,437-38,584,533	1125096	p13.2 - p13.1	CN Loss	24
chr9:71,809,604-72,115,761	306157	q21.11	CN Gain	7
chr9:72,553,204-72,672,000	118796	q21.11	CN Gain	3
chr9:73,297,024-73,509,447	212423	q21.13	CN Gain	2
chr9:74,218,987-76,262,573	2043586	q21.13	CN Gain	3
chr9:76,815,681-77,997,054	1181373	q21.13	CN Gain	5
chr9:80,415,779-87,415,705	6999926	q21.31 - q21.33	CN Gain	54
chr9:88,303,180-89,662,603	1359423	q21.33 - q22.1	CN Gain	12
chr9:100,184,507-101,615,735	1431228	q22.33 - q31.1	CN Gain	36
chr9:102,809,552-106,659,534	3849982	q31.1	CN Gain	28
chr9:107,640,801-109,047,049	1406248	q31.2	CN Gain	5
chr9:109,878,226-112,881,401	3003175	q31.2 - q31.3	CN Gain	30
chr9:113,540,171-125,646,970	12106799	q31.3 - q33.2	CN Gain	149
chr9:126,122,060-126,384,399	262339	q33.3	CN Gain	7
chr9:129,962,664-130,803,316	840652	q34.11	CN Loss	39
chr9:130,878,302-131,284,406	406104	q34.11	CN Loss	9
chr9:131,590,678-131,662,639	71961	q34.11	CN Loss	5
chr9:134,915,735-140,037,581	5121846	q34.2 - q34.3	CN Loss	210

Supplementary Table S2. Genes residing within the differentially lost, aggressiveness-associated 9p21.3

chr9:21,340,769-21,921,880 (Region length 581,111)							
**Gene Symbol**	**Chromosome**	**Start**	**End**	**Name**	**Biological Process**	**Cellular Component**	**Molecular Function**
IFNA6	chr9	21340316	21340886	*interferon, alpha 6*	defense response, response to virus	extracellular region, extracellular space	cytokine activity, cytokine receptor binding
IFNA13	chr9	21357370	21358075	*interferon, alpha 13*	defense response, response to virus	extracellular region, extracellular space	cytokine activity, interferon-alpha/beta receptor binding
IFNA2	chr9	21374254	21375396	*interferon, alpha 2*	cell surface receptor linked signal transduction, cell-cell signaling, induction of apoptosis, inflammatory response, response to virus	extracellular region, extracellular space	cytokine activity, interferon-alpha/beta receptor binding
IFNA8	chr9	21399145	21400184	*interferon, alpha 8*	defense response, response to virus	extracellular region, extracellular space	cytokine activity, cytokine receptor binding
IFNA1	chr9	21430439	21431315	*interferon, alpha 1*			
IFNE	chr9	21470840	21472312	*interferon, epsilon*	defense response, response to virus	extracellular region, extracellular space	cytokine activity, cytokine receptor binding
LOC554202	chr9	21444266	21549697	*hypothetical LOC554202*			

(*Continued*)

Gene Symbol	Chromosome	Start	End	Name	Biological Process	Cellular Component	Molecular Function
MTAP	chr9	21792634	21855969	*methylthioadenosine phosphorylase*	nicotinamide riboside catabolic process, nucleobase; nucleoside; nucleotide and nucleic acid metabolic process, nucleoside metabolic process	cytoplasm	S-methyl-5-thioadenosine phosphorylase activity, phosphorylase activity
**chr9:21,997,056-22,196,966 (199,910)**							
CDKN2B	chr9	21992901	21999312	*cyclin-dependent kinase inhibitor 2B (p15, inhibits CDK4)*	G1/S transition checkpoint, G2/M transition of mitotic cell cycle, cell cycle, cell cycle arrest, cellular response to extracellular stimulus, cellular response to nutrient, negative regulation of epithelial cell proliferation, negative regulation of phos	cytoplasm	cyclin-dependent protein kinase inhibitor activity, protein binding, protein kinase binding
CDKN2BAS	chr9	21984789	22111091	*CDKN2B antisense RNA (non-protein coding)*			

Supplementary Table S3. Univariate survival analysis

Parameters	Category	No. of patients with follow-up	DSS		MeFS	
			No. of event	*p*-value	No. of event	*p*-value
**Sex**	Male	47	10	0.9720	13	0.3750
	Female	36	6		11	
**Age**	<60 years	30	6	0.6483	7	0.1755
	≧60 years	53	10		17	
**Location**	Extremity	63	11	0.1758	17	0.2575
	Axial	20	5		7	
**Tumor size**	<6 cm	39	5	0.2962	6	0.0126*
	≧6 cm	38	7		14	
**FNCLCC grade**	Grade 1	35	2	0.0160*	3	0.0011*
	Grade 2 and 3	48	14		21	
**AJCC stage**	Stage 1 and 2	48	6	0.0813	9	0.0130*
	Stage 3	32	8		13	
**MTAP protein**	Expression	54	6	0.0195*	11	0.0572
	Deficiency	29	10		13	
**MTAP protein#**	Expression	46	4	0.0129*	8	0.0150*
	Deficiency (*HD*)	20	8		11	
	Deficiency (*PM*)	8	1		2	
	Deficiency (*no HD or PM detected*)	2	0		0	

*Statistically significant *p*-values; DSS, disease-specific survival; MeFS, metastasis-free survival, HD: homozygous deletion, PM: promoter methylation

#Statistical analysis in 76 cases with both informative *MTAP* gene status and follow-up information

Supplementary Table S4. Multivariate survival analysis

Outcome	Variable	Category	RR	95% CI	*p*-value
**DSS**	**MTAP**	Expression	1.000	-	0.0140*
		Deficient	4.020	1.326-12.194	
	**FNCLCC grade**	Grade 1	1.000	-	0.0688
		Grade 2 and 3	4.754	0.890-23.516	
	**AJCC stage**	Stage 1 and 2	1.000	-	0.5707
		Stage 3	1.395	0.441-4.411	
**MeFS**	**FNCLCC grade**	Grade 1	1.000	-	0.0201*
		Grade 2 and 3	4.804	1.278-18.055	
	**MTAP**	Expression	1.000	-	0.0318*
		Deficient	2.527	1.084-5.893	
	**AJCC stage**	Stage 1 and 2	1.000	-	0.3692
		Stage 3	1.527	0.606-3.843	

*Statistically significant; RR, risk ratio; CI, confidence interval

Supplementary Table S5. Differentially expressed angiogenesis-associated genes selected from the RT-PCR expression array

Gene Symbol	Ref. Seq	Description	Fold Regulation	*p*-value
CXCL10	NM_001565	Chemokine (C-X-C motif) ligand 10	-8.5436	<0.000001
CXCL9	NM_002416	Chemokine (C-X-C motif) ligand 9	-12.1208	0.000012
FGFR3	NM_000142	Fibroblast growth factor receptor 3	-24.3805	<0.000001
FLT1	NM_002019	Fms-related tyrosine kinase 1 (vascular endothelial growth factor/vascular permeability factor receptor)	-17.1521	0.000004
MMP9	NM_004994	Matrix metallopeptidase 9 (gelatinase B, 92kDa gelatinase, 92kDa type IV collagenase)	-24.3007	<0.000001
TNF	NM_000594	Tumor necrosis factor	-17.8008	0.000003

Supplementary Table S6. Associations of MTAP expression with proliferative index, microvessel density, and MMP-9 expression level

	MTAP Expression (n=40)		p-value
	Deficient (n=17)	Positive (n=23)	
Ki-67 LI (range, 0~100)	51.8±17.58	21.5±15.84	P<0.001*
MVD (mm^2)	77.5±27.85	50.6±22.45	P=0.002*
MMP-9 H-score (range, 100~400)	270.9±47.34	201.6±39.14	P<0.001*

LI: labeling index,
*Statistically significant by Mann-Whitney U Test

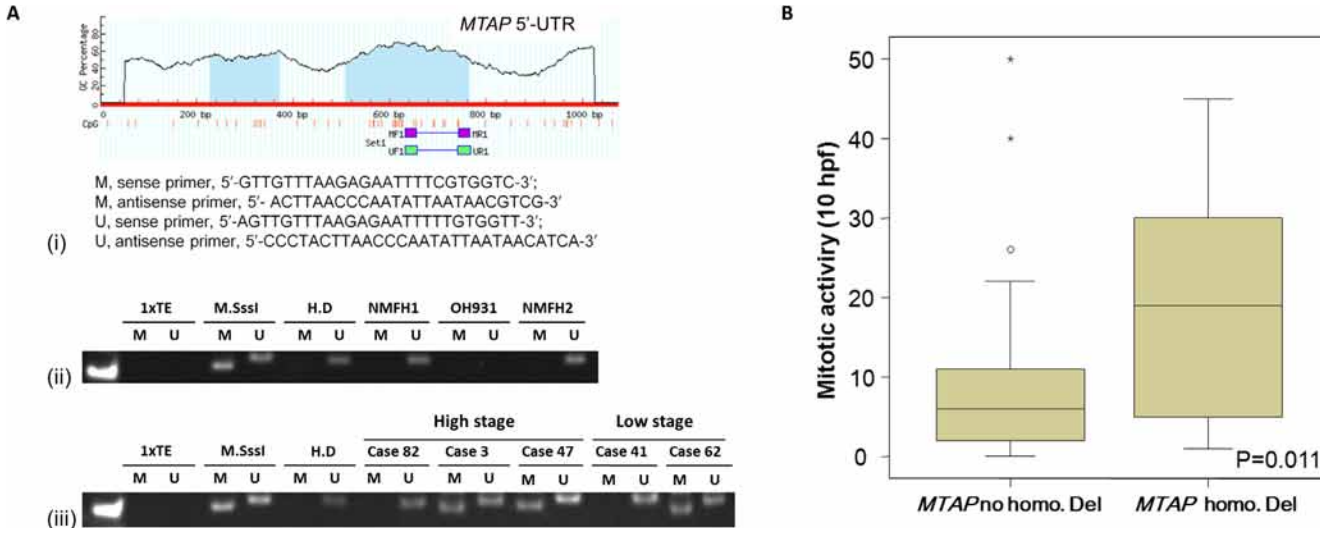

Supplementary Figure S1: (A) Methylation-specific PCR targeting a *MTAP* promoter. Methylation-specific PCR targeting of the 5' regulatory element of MTAP exhibited no methylated band in the MTAP-homozygously deleted OH931 or NMFH-2 cell lines or the MTAP-expressing NMFH-1 cell line. In representative formalin-fixed tumor specimens, MTAP promoter methylation can be distinctly visualized in myxofibrosarcomas at the low (Case 62) and high stages (Case 3, Case 47). (TE, TE buffer; M.SssI, M.SssI methyltransferase-treated DNA; HD, a healthy donor's DNA without treatment of M.SssI methyltransferase; M, methylation-specific primers; U, unmethylation-specific primers). **(B)** Comparison of mitotic activity between myxofibrosarcomas with and without homozygous deletion. A significantly high count in the MTAP was observed in the homozygously deleted myxofibrosarcomas.

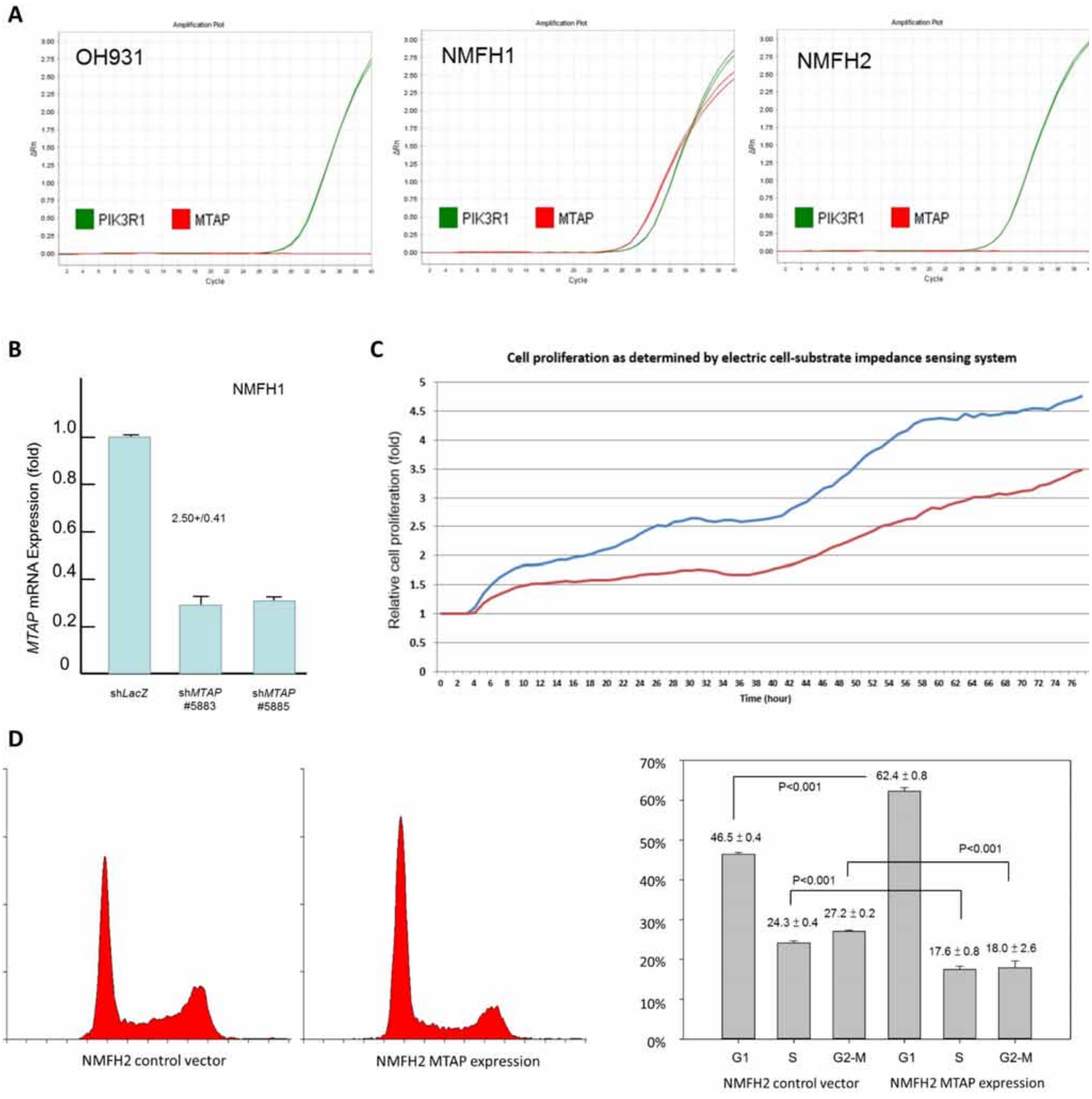

Supplementary Figure S2: (A) Determination of *MTAP* gene dosage by using real-time PCR. The quantitative PCR for MTAP genomic DNA is shown in the representative amplification curves of the myxofibrosarcoma cell lines, confirming the homozygous deletion of MTAP in the OH931 (left) and NMFH-2 (right) cells, but exhibiting the detectable MTAP gene in the NMFH-1 cells (middle). PIK3R1 serves as the reference gene. **(B)** shRNA-mediated knockdown of endogenous MTAP mRNA expression. By using quantitative RT-PCR, 2 clones of shMTAP were observed to successfully knock down the endogenous MTAP mRNA in the NMFH-1 cells. **(C)** ECIS assay. The cell number detected in a real-time manner significantly decreased in the MTAP-reexpressing NMFH-2 cells, compared with the empty control. **(D)** Flow cytometric assay to evaluate cell cycle kinetics. Compared to the empty control, MTAP-reexpressing NMFH-2 cells exhibited significant cell cycle arrest in the G_1 phase (left), which significantly increased from 46.5% to 62.4% ($p < 0.001$), accmopanined by concomitantly decreased percentages of cells in the S and G_2 phases as illustrated in the histograms (right).

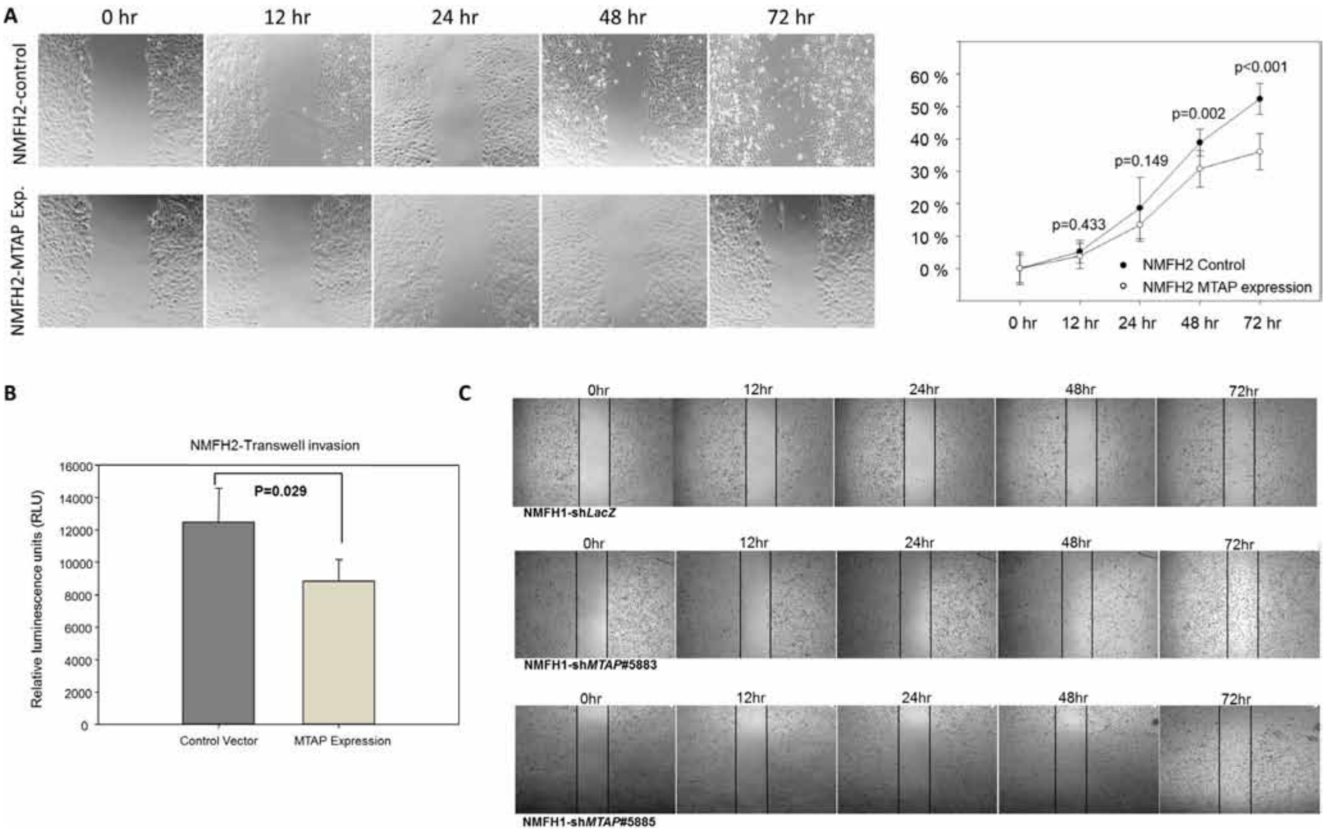

Supplementary Figure S3: MTAP attenuates myxofibrosarcoma cell migration and invasiveness. (A, C) The wound closure monitored for 72 h was significantly enhanced in NMFH-2 cells transfected with empty vectors **(A)** and NMFH-1 cells transfected with shMTAP **(C)** compared with the corresponding counterparts transfected with MTAP-expressing plasmids and shLacZ. **(B)** In the transwell invasion assay, the number of empty vector-transfected NMFH-2 cells invading through the matrigel-coated membrane was significantly increased.These results indicated that the MTAP inhibits the migratory and invasive capabilities of myxofibrosarcoma cells. The experiments were performed in triplicate, and the results are expressed as mean ± SD.

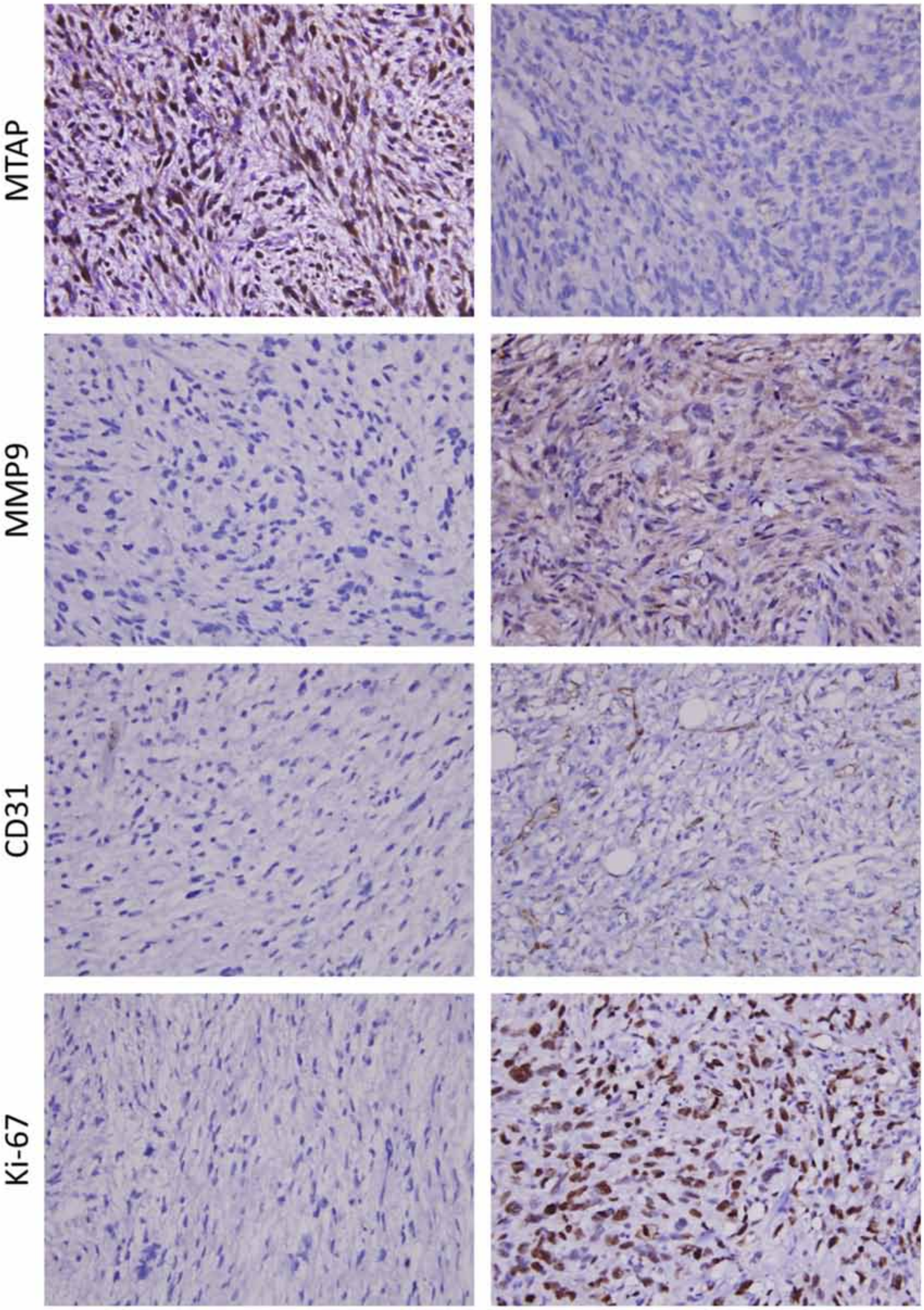

Supplementary Figure S4: MTAP protein deficiency is associated with increased expression of MMP-9, CD31-labeled microvessel density, and Ki-67 labeling index in primary myxofibrosarcoma tissues. In contrast to the representative MTAP-preserved myxofibrosarcoma *(left column)*, the MTAP-deficient tumor sample *(right column)* significantly increases expression of MMP-9, CD31-labeled endothelial cells, and Ki-67.

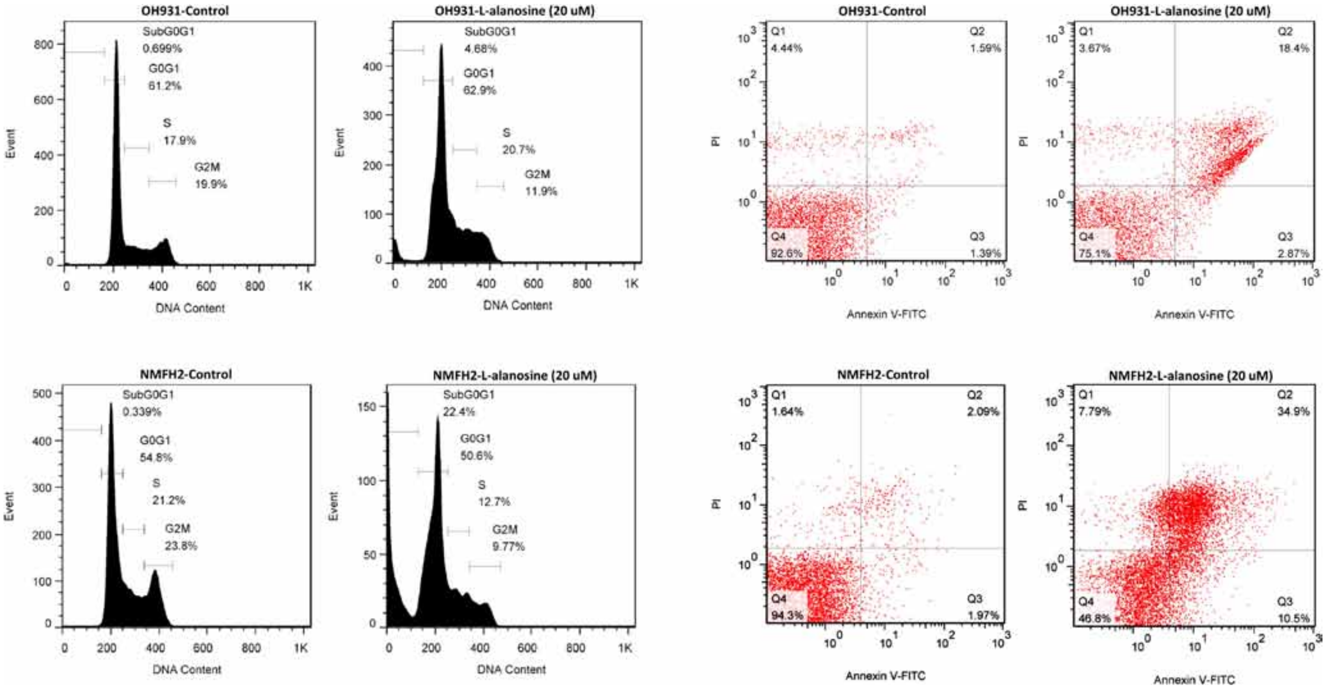

Supplementary Figure S5: L-alanosine induces apoptosis in MTAP-deficient myxofibrosarcoma cells. (Left) In a flow cytometric analysis, a significantly increased sub-G_1 population was detected in both L-alanosine-treated myxofibrosarcoma cell lines, and was particularly prominent in the NMFH-2 cells compared with the corresponding vehicle controls. **(Right)** Following annexin V-FITC and propidium iodide staining, the cell percentages at the early (Annexin V [+], PI [-]) and late (Annexin V [+], PI [+]) stages of apoptosis are shown as mean ± SEM, indicating significant differences between the L-alanosine-treated OH931 and NMFH-2 myxofibrosarcoma cells and their corresponding vehicle controls.
